# 

**Published:** 1916-06-03

**Authors:** 


### CONTENTS.

| | PAGE |
|---|---|
| **Organisation in the Medical Profession** ... | 201 |
| **Epsom College** ... ... ... ... ... ... | 202 |
| **Hospital and Institutional News** ... ... ... | 203 |
| Sir Douglas Haig's Dispatches—Prevention of Sickness Recognised—Women and Cambridge University Medical Examinations—The Wounded and the London Squares—The General Medical Council—A Medical School for the Transvaal—Hospital Aid from the United States—Naval Hospital Standard—The Duke of Rutland's Double Warning—A London Hospital Mystery—The City and the Royal Free Hospital—Medical Gymnastics at Huddersfield—The Samaritan Society in War-time—The Woman Doctor in the Dark—A Reserve Staff—Paper-saving at St. Mary's Hospital—Scottish War Hospital Activities—A Hospital Arbitration—The Ambitious Gazette—A Reward for Cottage Hospital Training—A New Tuberculosis Order—Assistance for Tuberculous Soldiers—The Metropolitan Asylums Board and its Employees—Nurses and the National Poor-Law Officers' Association—The Royal Commission on Venereal Diseases—The Detention of Cases—The Belfast Guardians and their Medical Officers—Contracts Between Guardians and Medical Officers—The Medical Officer's Salary at Driffield—This Week's Drug Market. | |
| **Military Amputations** ... ... ... ... ... | 209 |
| II.—The Treatment of Stumps in Preparation for Artificial Limbs. | |
| **Foods, Fats, and Feeding** ... ... ... ... | 210 |
| **A Great Physician and Gentleman** ... ... | 211 |
| Sir James Frederic Goodhart, Bart., M.D. Aberdeen, F.R.C.P. Lond. | |
| **A Medical Causerie** ... ... ... ... ... | 214 |
| **The Institutional Treatment of Consumption** | 215 |
| III.—Gradation of Work and Exercise. | |
| **Legal Notes for Institutional Workers** ... | 216 |
| Liability of Hospital and School Managers Compared—Fees of Doctors as Witnesses. | |
| **King Edward's Hospital Fund for London** ... | 217 |
| Annual Meeting of Governors and General Council. | |
| **Provision for Disabled Soldiers at Cardiff** ... | 219 |
| **Hospital Results in 1915** ... ... ... ... | 220 |
| **Passing Events and Topics** ... ... ... | 220 |
| **The Matrons' and Sisters' Department** ... | 221 |
| Household Management: The Little Store Snare—The Feeding of Hospital Staffs. | |
| Round the Hospitals. | |
| Qualifications for the College Register—Dietetics in Military Hospitals—Soldier Patients: Dangers of Premature Discharge—Miss Nightingale: Curious Record. | |
| **The Institutional Library** ... ... ... ... | 223 |
| **The Editor's Letter-Box** ... ... ... ... | iv |
| The Adequate Feeding of Patients and Staff. | |
| **The English of Medical Writings** ... ... | iv |
| **The Institutional Worker** ... ... (Suppl.) | 1 |
| Co-operative Poultry Farming for Hospitals. Question for June. | |

